# Comparison of Commercial and Experimental Fibre-Reinforced Composites in Restoring Endodontically Treated Teeth with Minimal Coronal Dentine: An In Vitro Study

**DOI:** 10.3390/jfb16090335

**Published:** 2025-09-08

**Authors:** Amre R Atmeh, Faisal Masaud, Luba AlMuhaish, Abdulkarim Alanazi, Hadeel Almutiri, Saqib Ali, Hassan Almoqhawi, Abdul Samad Khan

**Affiliations:** 1 Hamdan Bin Mohammed College of Dental Medicine (HBMCDM), Mohammed Bin Rashid University of Medicine and Health Sciences (MBRU), Dubai P.O. Box 505055, United Arab Emirates; amratmeh@yahoo.com; 2 College of Dentistry, Imam Abdulrahman Bin Faisal University, Dammam P.O. Box 1982, Saudi Arabia; faisal.s.masaud@gmail.com (F.M.); luba.almuhaish@gmail.com (L.A.); kalanazi98@gmail.com (A.A.); hadeelmutiri@hotmail.com (H.A.); 3 Department of Biomedical Dental Sciences, College of Dentistry, Imam Abdulrahman Bin Faisal University, Dammam P.O. Box 1982, Saudi Arabia; samali@iau.edu.sa; 4 Department of Restorative Dental Sciences, College of Dentistry, Imam Abdulrahman Bin Faisal University, Dammam P.O. Box 1982, Saudi Arabia; almoqhawi.h.a@gmail.com

**Keywords:** endocrowns, endodontically treated teeth, direct restorations, coronal dentine, fracture strength, hydroxyapatite grafted composite, fibre-reinforced composite

## Abstract

Aim: To compare the fracture resistance of teeth with varying degrees of residual coronal dentine after restoration using two fibre-reinforced composite core materials. Materials and Methods: Seventy extracted human lower premolars were divided into four groups: sound (control), one missing proximal wall (Cl-II), two missing proximal walls (MOD), and endocrown (EC). Subgroups were restored with either a short fibre-reinforced flowable composite (EverX Flow) or an experimental fibre-reinforced composite. Except for the control, teeth underwent endodontic treatment and were restored accordingly. Fracture resistance was tested using a universal testing machine. Statistical analysis compared fracture resistance across groups. Results: Teeth in EC exhibited the highest fracture resistance (1153.43 ± 332.52 N), comparable to sound teeth (1114.03 ± 185.58 N) and not significantly different from the experimental composite group (1006.89 ± 200.51 N) (*p* = 0.304). Cl-II restorations with EverX had significantly lower strength (652.48 ± 314.04 N) compared to MOD (773.02 ± 261.18 N) and EC (*p* < 0.05). The experimental composite showed a similar trend, with MOD having the lowest strength (408.6 ± 168.85 N). Significant differences were noted between materials in the MOD group (*p* = 0.009). Scanning electron microscopy revealed distinct fracture patterns. Conclusions: Endocrowns using direct fibre-reinforced composites provided protection for endodontically treated teeth with higher fracture resistance compared to teeth with MOD and Cl-II cavities. This gives direct composite endocrowns a potential for high-stress applications, though design and material selection remain critical.

## 1. Introduction

While root canal treatment aims to treat or prevent apical disease, the ultimate goal of the treatment is to maintain the longevity and function of the dentition. Due to the substantial loss of coronal hard tissues associated with root canal treatment, teeth could become more susceptible to fracture [1]. Dentine loss due to caries, access cavity preparation, and previous trauma may all contribute to the amount of remaining dentine. The fracture resistance of endodontically treated teeth could be further impacted by using various chemicals and intracanal medications [2] and decreased proprioception during function [3]. Therefore, a protective cuspal coverage restoration is fundamental for root canal-treated teeth with considerable loss of coronal dentine [4].

In cases where there is minimal remaining coronal dentine, the placement of an indirect restoration, such as a full crown, can lead to additional dentine loss. Such restorations may not be viable if preparing the tooth results in the loss of coronal tissues, ultimately rendering the tooth unrestorable, despite the integrity of the roots and surrounding bony structures. In these instances, a direct composite restoration with cuspal coverage, which requires no tooth preparation, presents a valuable solution and serves as a cost-effective treatment option [5]. Recent advancements in dental composites and adhesive systems have made this approach increasingly feasible. Newly introduced fibre-reinforced composite materials have been shown to enhance the strength of compromised teeth, thereby reducing the risk of fracture in root canal-treated teeth [6,7,8]. These composites were associated with enhanced mechanical retention, which mitigated the spread of fractures and established a robust bond between the glass fibres and the resin matrix [9].

Fibre-reinforced composites are produced by enclosing glass fibres in a resin matrix, while silane coupling agents chemically link these fibres to a polymerised monomer matrix [10]. The fillers help distribute stresses from the polymers to the fibres and prevent the initiation and propagation of cracks [11]. Fibre-reinforced composites have many benefits, including higher resilience, suitability for chair-side handling, and the ability to be customised to meet the specific needs of dental applications [7,12]. Moreover, the fibres play an essential role as they shift the stresses from an applied load from the weaker resin matrix to the stronger fine fibres. Studies conducted by Abdulmajeed et al. and Tsue et al. provided empirical findings substantiating the significance of correctly selecting the composite’s fibre type, length, diameter, and orientation [13,14]. It is reported that fibre-reinforced composites had higher storage modulus, flexural modulus and strength than composite resins with particulate filler [11,15]. The short glass fibres tend to reduce the polymerisation shrinkage, have higher aesthetic properties, adhere to the resin matrix, and are covalently combined three-dimensionally within the resin matrix [16,17].

EverX Flow is a short fibre-reinforced flowable composite used for dentine replacement and core build-up in dental restorations [18]. It is designed to flow and adapt to the cavity, providing both strength and fracture toughness attributed to fibre reinforcement. The incorporation of short E-glass fibres measuring 200–300 µm in length with a diameter of 6 µm, along with particulate barium glass fillers, was found to enhance the material’s strength and fracture resistance, thereby minimising the risk of catastrophic failures [19]. In a recent study by Garoushi et al., EverX Flow exhibited the highest fracture toughness, flexural strength, and degree of conversion among other tested commercially available short fibre-reinforced composites [20]. Furthermore, in the same study, the crowns fabricated from EverX Flow exhibited the highest fracture resistance.

Recently, bioceramics materials have gained attention in restorative dental sciences, where bioceramics such as bioactive glass, amorphous calcium phosphate, and hydroxyapatite have been used in restorative dental materials [21,22]. Among these bioceramics materials, hydroxyapatite has higher mechanical properties and can be used in load-bearing areas [23]. The stoichiometric hydroxyapatite with a Ca/P of 1.67, which is close to dentine and bone, is one of the ideal materials for hard tissue regeneration and remineralisation. Previously, our group grafted nano-hydroxyapatite on surface-activated glass fibres in situ, and the physical, mechanical, and in vitro biological properties were investigated in detail [24,25,26,27]. The grafting of nano-hydroxyapatite on glass fibres showed potential to form an apatite layer at the dentine–restoration interface. However, it is important to understand the potential uses of this experimental composite as a core restorative material.

This study aimed to compare the fracture resistance of teeth with various degrees of residual coronal dentine after restoration using commercially available glass fibre-reinforced core composite and compare it with an experimental composite. The first null hypothesis proposes no differences in the fracture resistance of teeth restored with fibre-reinforced composites, regardless of the degree of remaining dentine. The second null hypothesis suggests no difference in the fracture resistance of restored teeth, irrespective of the type of core restorative material.

## 2. Materials and Methods

### 2.1. Sample Preparation

**Figure 1:**
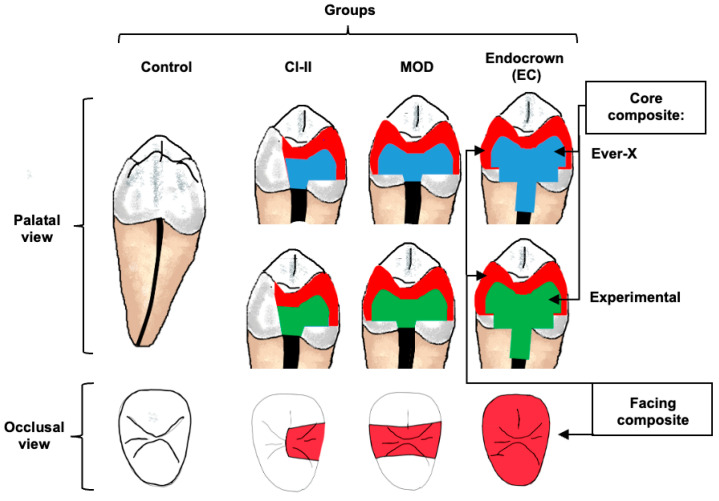
Group distribution of samples based on cavity design (Cl-II, MOD, Endocrown). A fibre-reinforced core composite (EverX or experimental) was used as a core material, which was faced by a posterior composite.

A power analysis (using G*Power version 3.1) was carried out for a one-way ANOVA with four groups, using a significance level of 0.05 and a power of 0.80. Based on our pilot fracture-resistance data, a large effect size (Cohen’s f = 0.41) was assumed, which suggested a minimum of 64 samples. To ensure balanced allocation and account for any potential specimen loss, we included 70 teeth (*n* = 10). Seventy healthy human lower premolars were collected after obtaining patients’ consent and appropriate ethical approval (IRB-2023-02-111). All teeth were extracted for clinical indications. Teeth were visually and radiographically inspected to exclude teeth with deep carious cavities, fractures or cracks, root resorption, curved roots, or unusual root/canal anatomy. The teeth were numbered and grouped according to their length. After that, stratified randomisation based on the length was applied to distribute the teeth into the groups. The distribution ensured maintaining the same ratio of teeth of the same length in each group. Teeth were distributed into three groups depending on the extent of coronal restoration involving one proximal wall (Cl-II), two proximal walls (MOD), and endo-crown (EC), Table 1. Teeth were subdivided into two subgroups depending on the material used for dentine replacement: short fibre-reinforced flowable composite (EverX Flow, GC, Tokyo, Japan) and an experimental fibre-reinforced composite (*n* = 10), in addition to a control group of sound teeth (*n* = 10) (Figure 1). Except for the control group, all teeth were endodontically treated. A single operator prepared the access cavities and prepared the canals chemo-mechanically using ProTaper^®^ Universal rotary instruments S1-F2 (Dentsply Maillefer, Ballaigues, Switzerland) with frequent irrigation using NaOCl (2%) solution. A final rinse with 17% ethylenediaminetetraacetic acid (EDTA) and 2% NaOCl was performed before obturation using a single gutta-percha cone (ProTaper F2) and epoxy resin-based sealer (AHPlus, Dentsply DeTrey GmbH, Konstanz, Germany).

### 2.2. Preparation of Experimental Composite

Initially, in situ nano-hydroxyapatite was grafted onto the surface of activated glass fibres using microwave irradiation techniques [Samsung MW101P, Suwon, Republic of Korea; 1000 W for 3 min (15 s ON: OFF)], as described in detail previously [28]. The concentration of glass fibres with nano-hydroxyapatite was optimised to 50 wt.%. The nano-hydroxyapatite grafted glass fibres obtained were heat-treated (Daihan Scientific, Wonju, Republic of Korea) at 450 °C with a heating rate of 10 °C/min. For the experimental composite, the nano-hydroxyapatite grafted glass fibres served as the reinforcing agent. All chemicals used to prepare the composite were purchased from Sigma Aldrich, St. Louis, MO, USA, and were of analytical grade. The dimethacrylate monomers, namely 2,2-bis [4-(2-hydroxy-3-methacrylyloxypropoxy)phenyl] propane (bis-GMA), triethylene glycol dimethacrylate (TEGDMA), and urethane dimethacrylate (UDMA), were initially mixed for 60 min, with their ratios optimised to 40:25:35, respectively. The weight percentages of each monomer were calculated based on these ratios. After mixing the resin monomers, photoinitiators (camphorquinone and Ethyl 4-(dimethylamino) benzoate) were added, each at a concentration of 0.5 wt.%. The resin mixture was stirred for 30 min in a dark environment at room temperature. Subsequently, 50 wt.% of nano-hydroxyapatite-grafted glass fibres was added incrementally and stirred overnight at room temperature in the dark to prevent premature polymerisation [29]. Following preparation, the experimental composite was stored in a dark bottle at refrigerated temperature until further use.

**Figure 2:**
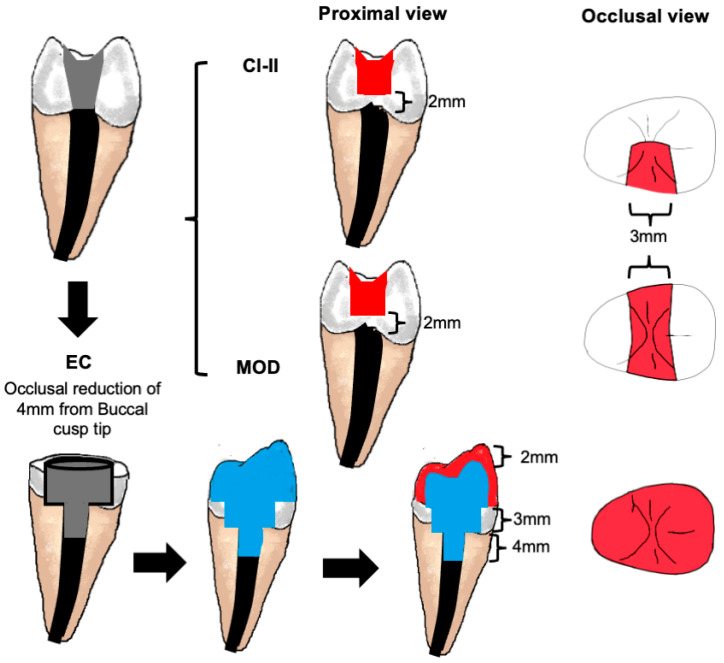
Cavity preparation and restoration following endodontic treatment in different groups. In the Cl-II and MOD groups, the endodontic access cavities were extended proximally to involve one or both proximal walls, respectively, with the gingival floor 2 mm above the cemento-enamel junction. In the EC group, after occlusal reduction, the remaining coronal tissue thickness was reduced to 1.5 mm at a depth of 3 mm. A Nayyar intracanal post space of 4 mm was prepared. A fibre-reinforced composite was used as a core material, which was faced by a posterior composite.

Teeth were prepared by a single operator and restored according to their groups (Table 1). In groups (Cl-II) and (MOD), the access cavities were extended to one or both proximal surfaces, respectively, by cutting the hard dental tissues to a level 2 mm above the CEJ to create 3 mm wide proximal extension(s) (Figure 2). In the (EC) group, the coronal enamel and dentine were reduced from the cusp tip by 4 mm, and the endodontic access cavity was extended circumferentially above the CEJ level, leaving a thickness of 1.5 mm of outer hard dental tissues that was verified using a calibre dental ruler. A Nayyar post space was also prepared by removing the root filling material 3 mm below the CEJ level using a standardised round bur size. Before preparation, two occlusal silicone indices were recorded for the crown of a representative tooth in this group using a putty silicone impression material (Express XT Putty, 3M ESPE). One index was cut in the middle as a reference to ensure sufficient occlusal reduction by 4 mm, leaving enough thickness for the core and facing composite materials placed in 2 mm thicknesses each. The other putty index was used as a mould for the facing composite material after core/post build-up.

A universal composite restorative material (Filtek Z250 3M ESPE, Seefeld, Germany) was applied at a 2 mm thickness facing for the core materials. All composite materials were applied as per the manufacturer’s instructions after 15 s of acid etching of the hard dental tissues using 37% phosphoric acid gel, followed by water rinsing for 30 s and application of a single bottle bonding agent (Adper™ Single Bond Plus Adhesive, 3M ESPE, Seefeld, Germany) before light curing using a light emitting diode (LED) light intensity ~800 mW/cm^2^; wavelength ~430–490 nm; Ivoclar Vivadent LEDition, Schaan, Liechtenstein, Austria) light cure for 40 s.

### 2.3. Testing of Fracture Strength

#### 2.3.1. Two-Body Wear Test—Chewing Simulation

The teeth samples were mounted in the acrylic mould to maintain equal height. The apical 5 mm root surfaces were coated with a thin layer of polyvinylsiloxane impression material (Dentsply Aquasil, Milford, DE, USA) ranging from 0.2 to 0.3 mm in thickness, to simulate a periodontal membrane using a microbrush. The chewing simulation (120,000 cycles; Chewing simulator; CS-4.2 SD Mechatronik GmbH, Feldkirchen-Westerham, Germany) was performed prior to the fracture resistance test. The vertical and horizontal movement of the antagonist was set at 2.0 mm and 0.7 mm, respectively, whereby the vertical speed was set at 40 mm/s and load weight was 20 kg, equivalent to an effective loading force of approximately 200 N.

#### 2.3.2. Fracture Resistance Test

A universal testing machine (Instron Model 5965, Norwood, MA, USA) was used, with a stainless-steel jig featuring a 4 mm diameter tip to apply vertical force over the restored cavity. The crosshead speed was 1 mm·min^−1^, and a slowly increasing vertical force was exerted until fracture occurred. The fracture moment was determined when a sudden drop in force occurred. The maximum force required to fracture each specimen was recorded in Newtons using the Bluehill Universal testing material software v 4.2.1 (Instron, USA). The fractured teeth were initially examined under a light microscope. (Luxo Microscope, Elmsford, NY, USA). Representative samples were gold-coated using a gold sputter (Quorum Technology, Lewes, UK) for 90 s, and the surfaces were examined using a scanning electron microscope (TESCAN VEGA 3, TESCAN, Brno, Czech Republic). The voltage was set at 10–20 kV, and images were taken at various magnifications.

### 2.4. Statistical Analysis

The mean and standard deviation values were used for statistical analysis using SPSS version 22 (IBM Software, Chicago, IL, USA). Normality of the data was checked using the Shapiro–Wilk test, and *p*-values greater than 0.05 indicated that the data were normally distributed. Two-way analysis of variance (ANOVA) was applied to evaluate the effect of restoration type or cavity design and their interaction, which was followed by post hoc Tukey’s test to identify statistically significant differences, when appropriate. Additionally, an independent sample *t*-test was used to compare the means between the control and each of the other experimental groups. A *p*-value less than or equal to 0.05 was considered statistically significant.

## 3. Results

**Figure 3:**
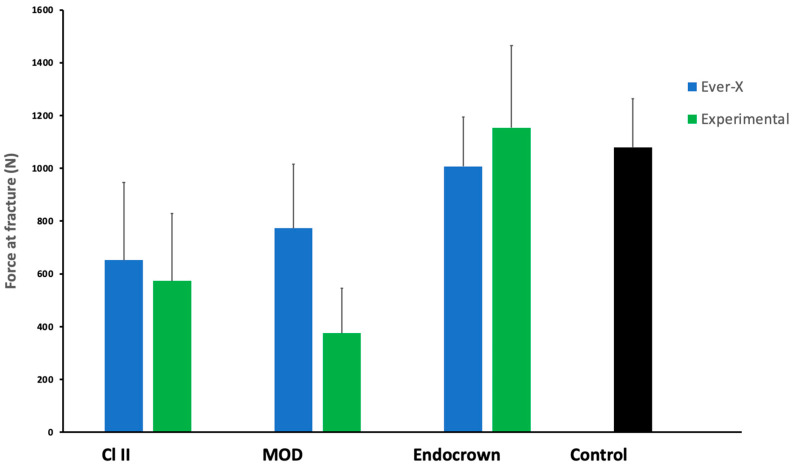
Fracture resistance of teeth with various degrees of residual coronal tissues after restoration with EverX and experimental (Exp) core materials.

The comparative fracture resistance results are shown in Figure 3. The mean fracture resistance ranged from 374.96 N (MOD–Experimental) to 1153.43 N (Endocrown–Experimental). Within the Class II group, EverX (652.48 N) had higher mean values than the Experimental material (573.53 N). In the MOD group, EverX (773.02 N) also exceeded the Experimental material 374.96 N). In contrast, in the Endocrown group, the Experimental material (1153.43 N) showed higher resistance than EverX (1006.89 N). The sound group recorded a mean of 1114.03 N.

The results of the two-way ANOVA, shown in Table 2, indicated no significant effect of the material type (*p* = 0.190) on the fracture resistance. In contrast, the effect of cavity design was found to be significant (*p* < 0.001), with a notable interaction between the two factors (*p* < 0.05). Among the different cavity designs, teeth restored with endocrowns showed the highest fracture resistance, which was significantly higher than the strength of teeth with Cl-II and MOD cavity designs.

The statistical data are tabulated in Table 3, showing the results of post hoc analysis comparing the different cavity designs within the same material type, and the *t*-test pair-wise analysis comparing the fracture strength of each group with the control group (sound tooth).

For the teeth restored with EverX, a significant difference (*p* < 0.01) was found comparing the three cavity designs. A lower mean fracture strength was observed in the teeth missing one proximal wall (Cl-II) (652.48 ± 314.04 N) compared to those with two missing walls (MOD) (773.02 ± 261.18 N); however, the difference was not statistically significant (*p* = 0.431). A non-significant difference (*p* = 0.080) was also found between the MOD and EC groups. On the contrary, the Cl-II and EC showed a significant difference (*p* = 0.019). Both Cl-II and MOD cavity designs restored with EverX exhibited a significant difference compared with the control group (sound tooth), with *p* values of 0.005 and 0.016, respectively. However, a non-significant difference (*p* = 0.305) was found between the EC and control groups.

For the teeth restored with the experimental composite, the lowest strength was found with the MOD design (374.96 ± 168.85 N) compared to the Cl-II (573.53 ± 273.68). However, a non-significant difference (*p* = 0.180) was found between both groups. Cl-II and MOD showed significant differences between the groups with the EC design, with *p*-values of 0.002 and 0.0001, respectively. Similarly, both Cl-II (*p* = 0.001) and MOD (*p* = 0.0001) showed significant differences with the control group, whereby a non-significant difference (*p* = 0.786) was found between EC and the control group.

Assessing the interaction effect of both independent variables confirmed significantly higher fracture strength of teeth restored with an endocrown using the experimental composite compared to teeth with Cl-II cavities restored with EverX (*p*= 0.010), and significantly higher fracture strength of teeth restored with an endocrown using EverX composite compared to teeth with Cl-II (*p* = 0.042) or MOD (*p* < 0.000) cavities restored with the experimental composite. Furthermore, teeth with MOD cavities restored with EverX exhibited significantly higher strength compared to those restored with the experimental composite (*p* = 0.19).

**Figure 4:**
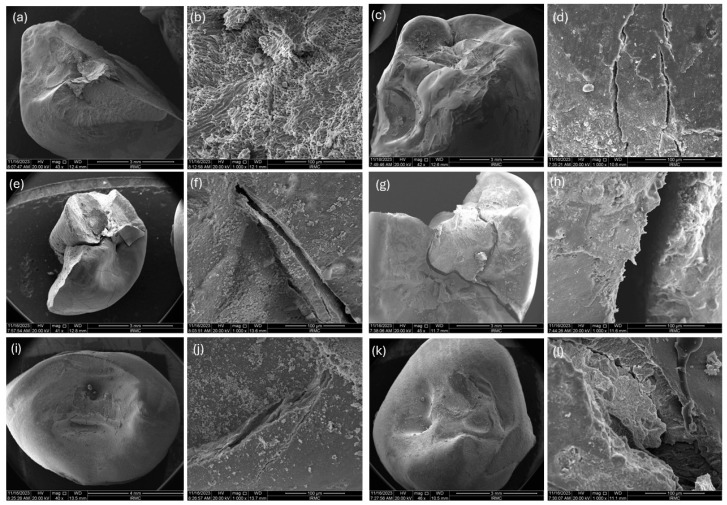
Fractographic images of Class II cavity design of (**a**,**b**) EverX, (**c**,**d**) Exp, Access cavity design of (**e**,**f**) EverX, (**g**,**h**) Exp; Endocrown design of (**i**,**j**) EverX, and (**k**,**l**) Exp.

The SEM micrographs presented in Figure 4 showed the fractured pattern of both groups with each cavity design. It was found that in the Cl-II cavity design for EverX, the restoration was removed entirely, and the tooth was fractured too. In contrast, for the experimental group, the restoration was partially removed. In the access cavity and endocrown designs, both restorations showed a similar pattern.

## 4. Discussion

This in vitro study evaluated the fracture resistance of endodontically treated teeth with various degrees of residual coronal hard tissues after restoration using two types of fibre-reinforced core composite materials. Comparing the fracture resistance between the different cavity design groups confirmed significant differences. Hence, the first null hypothesis can be rejected. Furthermore, comparing the effect of the core restorative material showed no significant differences between the two tested materials, hence the second null hypothesis is accepted.

The fracture resistance of endodontically treated teeth depends significantly on the amount of residual coronal tissues [30,31]. Therefore, the fracture resistance of teeth in Cl-II and MOD groups was significantly lower than sound teeth. Compared to endodontically treated teeth with Cl-II cavities, the survival rate of teeth with MOD cavities is lower due to the lesser amount of residual corona dentine, which leads to a greater cusp deflection [32]. However, no significant difference was found between the Cl-II and MOD groups for both materials. This may confirm the enhanced fracture resistance provided by the fibre-reinforced restorative materials, which demonstrated a greater effect on the MOD group. In a study comparing the survival of non-endodontically treated teeth restored with direct MOD fibre-reinforced composite restorations and indirect inlays, no significant difference in the cyclic load to failure was reported [33]. Improved fracture resistance of endodontically treated molars with MOD cavities was also reported when fibre reinforced bands or direct core materials were used with the composite restoration [34,35].

Although no effect of the restoration material was found on the fracture strength of teeth, regardless to the amount of residual coronal tissues, teeth with MOD cavity designs that were restored with EverX exhibited statistically significant higher strength than those restored with the experimental composite. This could be attributed to the different compositions and formulations of these composites, which was evident with weakest teeth. It is anticipated that the low value associated with the experimental group could be due to the manual preparation of the composite, whereas EverX is manufactured in a controlled environment. Furthermore, EverX contains short glass fibres and fillers (mainly barium glass), whereas the experimental group contains n-hydroxyapatite-grafted glass fibres and no additional fillers were added in the composition. The presence of barium glass might increase the fracture resistance of the restoration.

Compared to the EC group, teeth in both Cl-II and MOD groups exhibited significantly lower strength, although they had more residual coronal tissues. This could be partially related to the distribution rather than the amount of residual tissues. The cavity depth and thickness of residual tissues affected the fracture susceptibility of restored teeth [32,36]. Although the thickness of residual tissues was minimal in the teeth prepared for EC, the depth was higher in the Cl-II and MOD. Furthermore, preserving a band of intact coronal tissues in the EC group might have provided a protective mechanism to the endodontically treated teeth, similar to the ferrule effect. Although it had a reduced thickness, using a direct endocrown allowed the preservation of these tissues and prevented compromising the fracture resistance [37,38]. Another major factor is the reduced cuspal flexure associated with the cuspal coverage provided by EC. Covering the cusps can reduce cuspal flexure and fatigue and distribute the occlusal stress on a wider area [39].

In the teeth restored with EC, the fracture strength was comparable to that of the control group (sound teeth), with no significant differences between the two materials. As explained above, this may suggest that cuspal coverage with direct restorations as EC could be suitable for teeth with substantial loss of coronal tissues [38]. In a three-year prospective clinical study, the failure rate of endodontically treated teeth restored with direct composite onlays was comparable to similar teeth restored with crowns [40]. This may confirm that fibre-reinforced direct EC could offer a suitable restorative option for severely damaged teeth. This is primarily due to the lower cost of such treatment, which can prolong the tooth’s lifespan instead of necessitating extraction due to non-restorability. Unlike conventional crowns, the endocrown may not require preparation of the outer walls, as the available area provides sufficient retention, which is combined with adhesive bonding. This could provide a provisional solution until definitive indirect cuspal coverage restorations are placed [32]. Furthermore, for badly damaged teeth, such an approach could be the only possible option to save the tooth at a reasonable cost in case it fails.

Only lower premolars were used in this study, which may have more predictable results than molars when direct composite restorations are used [41]. Therefore, this could be a limitation of this study or a limitation in relating it to the performance of these materials in other types of teeth. Conducting this study in vitro meant that no accurate simulation of the forces and conditions inside the oral cavity was achieved. Although teeth were subjected to cyclic chewing loads before fracture strength testing, the test was applied in a static mode. This could overestimate the results obtained when compared to long-run clinical studies, where chewing involves dynamic, multi-directional loads that cannot be simply replicated with a unidirectional static load [42]. The lack of periodontal ligament is another limitation. Furthermore, the lack of other factors that could simulate the conditions inside the oral cavity, such as temperature and saliva, is also a limitation. Further investigations, including well-designed clinical studies, are still needed to validate and expand the clinical applications of fibre-reinforced composite core materials in restoring compromised endodontically treated teeth, which could be a cost-effective treatment option in restoring compromised teeth.

## 5. Conclusions

Within the limitations of this in vitro study, it can be concluded that restoring endodontically treated teeth with direct endocrowns using fibre-reinforced composites could be a valid option to consider. This could be of great value in situations where costly indirect restorations need to be avoided due to financial constraints or questionable tooth survival. Further clinical studies are needed to verify these findings.

## Figures and Tables

**Table 1:** Group distribution and description of cavity and restoration designs.

Group	Extent of Residual Coronal Dental Tissues	Restoration Design
Control	Sound tooth	None
Cl-II	Access cavity + 1 missing proximal wall.	Core: Fibre-reinforced composite Facing: Occlusal + 1 proximal wall with conventional posterior composite
MOD	Access cavity + 2 missing proximal walls.	Core: Fibre-reinforced composite Facing: Occlusal + 2 proximal walls with conventional posterior composite
EC	Minimal remaining coronal dental tissues.	Core + 3 mm Nayyar post: Fibre-reinforced composite Facing: Full coverage with conventional posterior composite

**Table 2:** Results of Two-way ANOVA.

Variables	Sum of Squares	df	Mean Square	F	Sig.
Type of material	125,967.588	1	911,284.639	1.77	0.190
Cavity design	2,559,628.470	2	838,041.046	18.01	0.000 *
Interaction	576,416.872	2	359,430.853	4.06	0.024 *

* Indicates statistically significant difference (*p* < 0.05).

**Table 3:** Comparison of means between different cavity designs and restorative materials.

	EverX	Exp	*p*-Value
Cl II	652.48 ± 314.04 ^a^*	573.53 ± 273.68 ^a^*	
MOD	773.02 ± 261.18 ^a,b^*	374.96 ± 168.85 ^a^*	0.190
EC	1006.89 ± 200.51 ^b^	1153.43 ± 332.52 ^b^	
*p*-value	0.014	0.0001	

Different lowercase superscript letters indicate statistically significant differences between the means in the same column based on the Two-way ANOVA and Tukey’s post hoc tests, with *p*-values presented for each material. Here, (*) indicates a statistically significant difference comparing the mean with the control group based on an independent sample *t*-test (*p* < 0.05).

## Data Availability

The original contributions presented in the study are included in the article, further inquiries can be directed to the corresponding author.
